# Biopanning data bank 2018: hugging next generation phage display

**DOI:** 10.1093/database/bay032

**Published:** 2018-03-27

**Authors:** Bifang He, Lixu Jiang, Yaocong Duan, Guoshi Chai, Yewei Fang, Juanjuan Kang, Min Yu, Ning Li, Zhongjie Tang, Pengcheng Yao, Pengcheng Wu, Ratmir Derda, Jian Huang

**Affiliations:** 1 Center for Informational Biology, University of Electronic Science and Technology of China, No. 2006, Xiyuan Ave, West Hi-Tech Zone, Chengdu 611731, China; 2 Department of Chemistry, University of Alberta, 11227 Saskatchewan Drive, Edmonton, AB T6G 2G2, Canada

## Abstract

The 2018 update of the biopanning data bank (BDB) stores phage display data sequenced by Sanger sequencing and next generation sequencing technologies. In this work, we upgraded the database with more biopanning data sets and several new features, including (i) incorporation of next generation biopanning data and the unselected population where the target is not determined and the round of screening is zero; (ii) addition of sequencing information; (iii) improvement of browsing and searching systems and 3 D chemical structure viewer; (iv) integration of standalone tools for target-unrelated peptides analysis within conventional phage display and next generation phage display (NGPD) data. In the current version of BDB (released on 19 January 2018), the database houses 3291 sets of biopanning data collected from 1540 published articles, including 95 NGPD data sets and 3196 traditional biopanning data sets. The BDB database serves as an important and comprehensive resource for developing peptide ligands.

**Database URL**: The BDB database is available at http://immunet.cn/bdb

## Introduction

Phage display allows the high throughput screening of phage-displayed libraries against multiple target molecules such as miRNAs (1), proteins (2, 3), polysaccharides (4), cells (5) and tissues (6). Typically, libraries with billions of peptides are subjected to iterative rounds of affinity selection, commonly referred to as biopanning (7), which rapidly enriches phage clones binding to the target of interest. Due to its high efficiency and versatility, screening of phage-displayed random peptide libraries has found wide applications in the development of diagnostics and therapeutics (8), drug-delivery reagents (9), biomaterials and inorganic functional materials (10, 11).

However, the identification of potential candidates after the biopanning process is a major challenge associated with the phage display technique. Traditionally, 20–100 individual clones from the final enriched population are randomly picked out and amplified. Then a target-binding screening assay can be used to distinguish the specific binders. Finally, the sequence of the displayed peptide with specific binding could be determined by Sanger sequencing. Given the vast diversity of phage-displayed peptide libraries (∼10^9^ unique sequences), Sanger sequencing analysis is low-throughput and provides a limited perspective (<0.01%) of the complete sequence space. More importantly, traditional phage display is badly hindered by the identification of notorious target-unrelated peptides (TUPs) (12, 13), which may dominate the small sample space and preclude the isolation of target-binding sequences. These TUPs can be divided into two types: selection-related (SrTUPs) and propagation-related (PrTUPs) (14). The selection of SrTUPs is caused by binding to non-target molecules in the biopanning system (15), whereas the isolation of PrTUPs is due to the advantage possessed by certain fast-propagating clones intrinsic to the library (12, 14, 16–18).

To overcome these challenges, many researchers have employed the phage display technology coupled with next generation sequencing (NGPD) in ligand identification (19–24), which allows for a comprehensive characterization of the sequence space of phage-displayed libraries. Next generation sequencing (NGS) technology has also been integrated with other *in vitro* selection techniques, such as messenger RNA (mRNA) display (25), yeast display (26) and aptamer selection (27). However, next generation phage display (NGPD) also possesses the challenge of evaluating which sequences are the most ideal ligands since the biopanning results are still a mixture of target-binding peptides and TUPs. To exclude any potential TUPs and identify peptides specifically binding to KS483 cells, ′tHoen and coworkers filtered all peptides with a frequency of 2 or higher in the naïve library and hits in PepBank and SAROTUP and selected target-related peptides from the remaining list of peptides (24). They also demonstrated that NGPD can promote the finding of specific binders by alleviating the problem associated with TUPs. Derda *et al.* have identified glycopeptide ligands for carbohydrate-binding proteins by comparing the frequency of each peptide after selection against the target molecule with the corresponding frequency for non-target molecules (28). As can be seen earlier, the strategy to determine target-related peptides can vary from researcher to researcher.

The popularity of the phage display technology inevitably led to the development of databases for phage display data. Artificially selected proteins/peptides database was the first database special for biopanning data (29), which incorporated biopanning data from 195 screening experiments. However, the database has not been upgraded since 2002. To gather these valuable data together, our group implemented and described the MimoDB database (MimoDB 1.0) for peptides selected from phage-displayed random peptide libraries (30). The updated MimoDB 2.0 includes biopanning data isolated from random peptide libraries constructed by diverse display technologies, including phage display, bacterial display, yeast display, ribosome display and mRNA display (31). The database, MimoDB, is actually the abbreviation of mimotope database. As the output of biopanning experiments contains both mimotopes and TUPs, the database was renamed as biopanning data bank (BDB) (32). There are other databases, such as PepBank (33), IEDB (34) and TumorHoPe (35), each harboring a part of biopanning data. These archives are not dedicated to biopanning data storage and take each unique peptide as an individual entry, whereas the result of biopanning usually consists of a set of peptides with different frequencies and affinities. However, it is a remarkable fact that all existing databases are exclusive of NGPD data.

Simultaneously, computational tools for analyzing biopanning data are continuously emerging. These programs can be employed to clean TUPs from the biopanning data (36–38) and interpret biopanning data (39, 40). Unfortunately, all these methods are only applicable to analysis of small-scale biopanning data obtained by traditional phage display. In recent years, powerful analytical methods have been designed for processing and translating sequences, including MATLAB-based single-end and paired-end conversion pipelines (41, 42). Furthermore, software for cluster or motif analysis within massive data has also been proposed, such as multiple specificity identifier (43), Hammock (44) and FASTAptamer (45). Additional methods have been built for finding potential target-binding ligands, such as Enrich2 for any counting-based enrichment/depletion Experiment (46) and PHASTpep for discovery of cell-selective peptides (47). Although these methods are successful in analyzing deep sequencing data, none of them can be utilized to perform TUP analysis within big biopanning data.

In this report, we describe a significant upgrade to the BDB database. We curated NGPD data from peer-reviewed publications and integrated standalone data analysis tools, which can facilitate TUP identification within traditional small scale biopanning data and ‘big biopanning data’ locally. The interactive structure viewer for target–template complex (TTC) or target–peptide complex (TPC) is re-implemented with JSmol and PHP. The search system has also been improved to be more user-friendly and intuitive. With traditional phage display and NGPG data, the BDB database will serve as an important and informational portal for biopanning data and a powerful evidence-based platform for the biopanning community to cross-check their panning results and ensure target-binding specificities. Thus, it provides a comprehensive resource for ligand identification-related studies. 

## Data upgrade

### Inclusion of NGPD and unselected data

**Figure 1.**
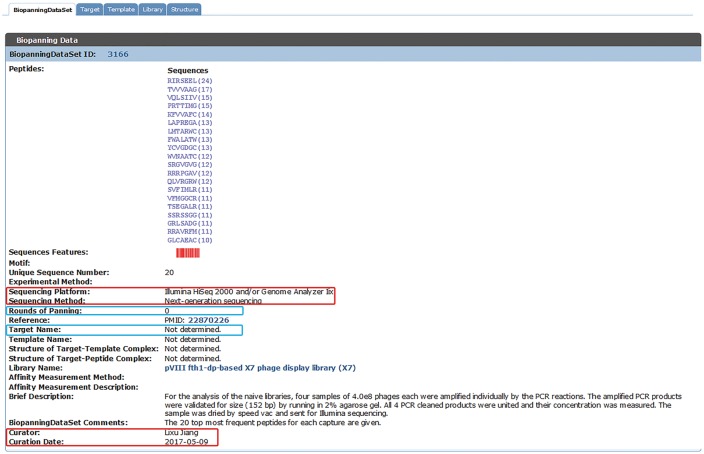
An example of a set of phage display data from the naïve library. New data fields are indicated by red boxes. For the unselected dataset, the corresponding target name is not determined and the round of panning is zero (highlighted by blue boxes).

As an updated version of BDB released in July 2015 (32) and MimoDB 2.0 (31), the current version of BDB complies with previous literature search methods, data collection approaches, data inclusion criteria and data organization style, which were described in great detail in the literature (31, 32). NGPD screens are more productive than traditional phage display selections, and NGPD data usually have millions of peptide sequences, whereas traditional phage display selections produce several hundreds of peptides. This presents a great challenge as well as an opportunity. In the current version of BDB (released on 19 January 2018), we curated 95 sets of NGPD data from 20 published papers. Sequencing data of selected populations and unselected libraries, for instance, the naïve library and the naïve library after one round or several rounds of amplification were collected. For the unselected dataset, the corresponding target name is not determined and the round of panning is zero (see Figure 1).

### New data fields

With the advance of sequencing technology, it is necessary to include sequencing information into BDB. For each entry of the biopanningdataset table, additional fields, including sequencing platform and sequencing method, were curated (see Figure 1, indicated by red boxes). According to our records, the BDB database stores peptide data sequenced by various high-throughput sequencing platforms, including Illumina Genome Analyzer^®^, Roche 454 GS System^®^, Ion Torrent system^®^ and Applied Biosystems ABI PRISM system^®^. However, most published articles using Sanger sequencing did not provide any information about sequencing platforms. Therefore, for most Sanger sequencing data sets, corresponding sequencing systems are unknown. We also added curation information, including curator and curation date (see Figure 1, marked by red boxes).

### Data summary and statistics

**Figure 2.**
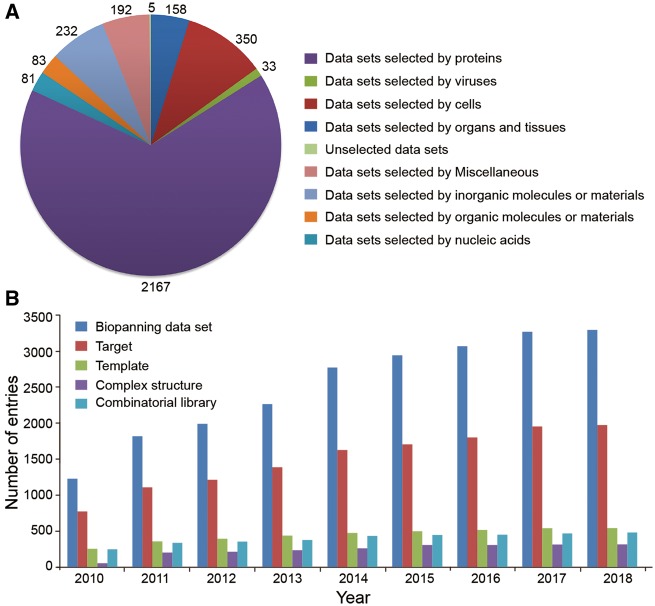
Summary of data entries in the BDB database. (**A**) Biopanning data sets are grouped into nine classes according to their target types. The number of biopanning data set selected by each type of target is shown. (**B**) Annual increase of data entries in BDB.

The BDB database released recently (19 January 2018) contains a total of 3291 sets of phage display data manually curated from 1540 peer-reviewed articles, a significant increase over the 2904 biopanning data sets in the previous major release (22 July 2015) (32). According to the sequencing technology, these data sets are distributed into 3196 sets of traditional biopanning data and 95 sets of NGPD data. Based on their target types, biopanning data sets can be divided into nine categories. As shown in Figure 2A, we can conclude that proteins, cells and inorganic molecules or substances are the three most commonly used targets in phage display screening experiments. Importantly, the current release of BDB also contains 29 795 peptides, 1971 targets, 545 templates, 484 different combinatorial libraries and 318 three-dimensional structures of TTC or TPC resolved experimentally (for instance, by X-ray crystallography or nuclear magnetic resonance spectroscopy). Generally, the BDB database is revised and updated once per quarter. Since its first release on 30 August 2010, the database has been updated as many as 29 times. We summarized the data entries increase annually (Figure 2B).

### Utilities upgrade

#### Browsing BDB

**Figure 3.**
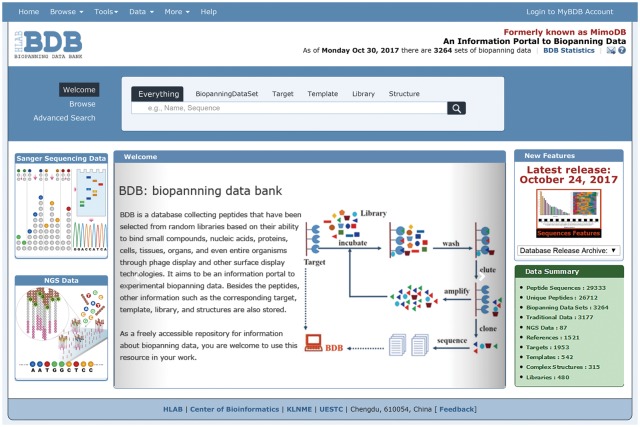
A screenshot of the BDB homepage. The homepage of BDB clearly shows that the BDB database contains both traditional and NGPD data. Users can browse these two kinds of data via clicking the corresponding figure on the left side of the website.

As described previously, each table in the BDB database can be quickly browsed via the ‘Browse’ drop-down menu or the ‘Browse’ option on the left side of the search panel (32). To improve user experience, we redesigned the BDB homepage layout. Currently, traditional or NGPD data can be browsed directly by clicking the corresponding figure on the left side of the BDB homepage (Figure 3). Thus users can conveniently visit these two types of data separately.

**Figure 4.**
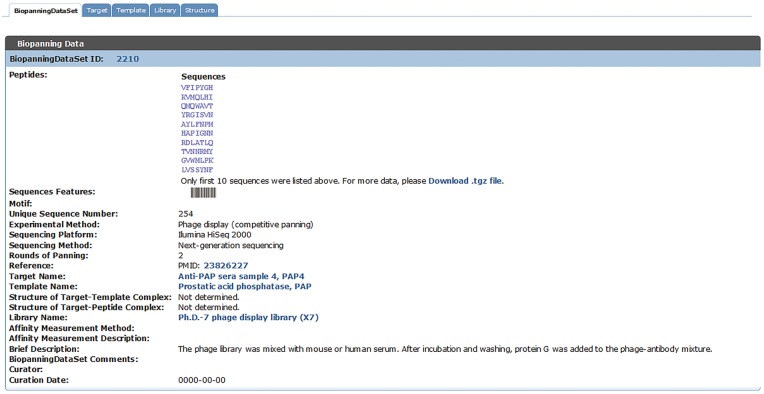
An example of a set of biopannning data with >200 peptides. If a set of biopanning data contains >200 peptides, the sequences features tool is disabled, and only the first 10 peptide sequences will be displayed and the complete data set is available for download via the provided link.

When browsing the biopanningdataset table, if the entry contains >200 peptide sequences, only the first 10 peptides will be displayed and the whole data set can be downloaded via the provided link (Figure 4). It is particularly worth mentioning that the on-the-fly data visualization tool, sequences features, is disabled when a set of phage display data contains <3 or >200 peptides (Figure 4).

#### Searching BDB

Based on user feedback, more data fields, such as sequencing platform, sequencing method, number of unique peptides, have been added to the advanced searching system. In the current release of BDB, most data fields in the database can be searched through the advanced searching system. For each data field to be searched, the search program will provide prompt and appropriate hint for users and help them quickly find data of interest, leading to an improved user experience.

#### Viewing complex structures

Java Applets have served as a primary program in displaying interactive 3 D chemical structures on a web page for about two decades. In fact, Jmol (Jmol: an open-source Java viewer for chemical structures in 3 D, http://www.jmol.org/) has been the 3 D viewer for the BDB website (30–32) and other web resources, such as the research (14) for structural bioinformatics protein data bank (RCSB PDB, http://rcsb.org) (48). Nevertheless, several Internet browsers (e.g. Chrome) no longer support Java Applets because of concerns for security precautions. In 2016, Oracle announced that Java Applets would be deprecated with Java version 9.0. To provide improved support for 3 D graphics, the BDB website has implemented JSmol (JSmol: an open-source HTML5 viewer for chemical structures in 3 D, http://wiki.jmol.org/index.php/JSmol) to interactively view TTC or TPC, which enables Jmol to display 3 D molecular structures on devices that do not have Java installed, or for which Java is not available or has not been installed.

#### Using BDB-powered tools

**Figure 5.**
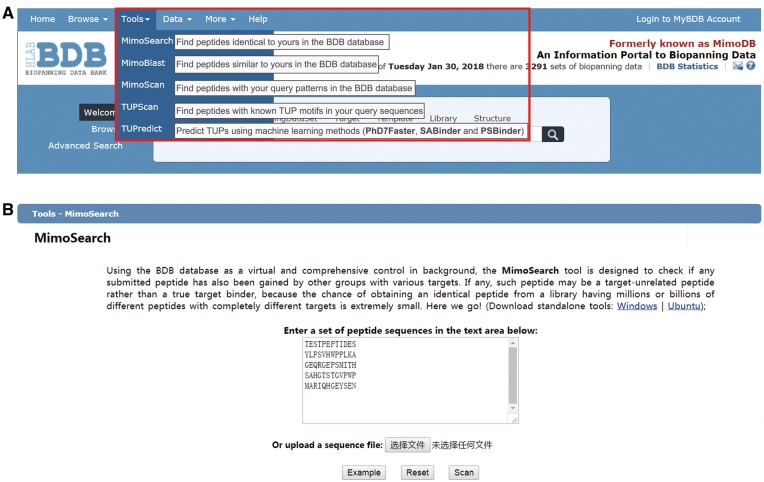
BDB-powered tools. (**A**) Seven tools surrounded by a red box, i.e. MimoSearch, MimoBlast, MimoScan, TUPScan, PhD7Faster, SABinder and PSBinder, can be accessible by clicking the secondary menu items from the ‘Tools’ drop-down menu. (**B**) We take MimoSearch as an example to show the utilization of these tools. Users can enter a set of peptide sequences in the text area or upload a sequence file. After submission, the result will be returned and displayed in a table.

In last major upgrade of BDB, we integrated a panel of tools in the SAROTUP suite into BDB (32). These tools can be classified into database-based, motif-based and machine learning methods-based tools and have played a significant role in cleaning TUPs from phage-displayed libraries and one-bead-one-compound combinatorial peptide libraries (49–52). However, these tools are unable to analyze next generation sequencing biopanning data. In the current release of BDB, we integrated standalone graphical user interface (GUI) and command-line versions for each tool developed using open source Qt 5.6 and C++ language, which can be used to find TUPs within biopanning results of conventional phage display and NGPD, whereas the web-based tools can be only employed to handle small-scale data. The offline version of each tool can run locally in Windows or Linux systems. The interface and utilization of the GUI version are similar to that of the web server. We also added a new machine learning method-based tool, known as PSBinder (53). This program allows users to predict putative polystyrene surface-binding peptides from biopanning data or to find novel candidates for polystyrene affinity tags. All tools can be available for download and free use. As shown in Figure 5A, six tools, i.e. TUPScan, MimoSearch, MimoBlast, PhD7Faster, SABinder and PSBinder, can be employed to perform TUP analysis, while MimoScan allows users to check how specific their consensus sequences, motifs or patterns derived from panning results are. We take MimoSearch as an example to show the utilization of these tools (Figure 5B). All these tools are valuable to the biopanning community. With the accumulation of more biopanning data in BDB, the database-based tools, including MimoSearch, MimoBlast, MimoScan, will be increasingly powerful and popular. We expect that cleaning TUPs from biopanning data will be adopted as a necessary and standard procedure in ligand development.

## Conclusion and future development

The BDB database is the largest archive for biopanning data, which provides a valuable resource for ligand development related studies. Currently, the database incorporates phage display data sequenced by both Sanger sequencing and high-throughput sequencing platforms. With the increasing popularity of NGPD, more sets of NGPD data will be added into the BDB database. The database will be continually maintained and updated. Furthermore, an experimentally supported dataset will be constructed and available in the near future, which will provide candidate peptides for drug and vaccine design and therapeutics and diagnostics development. Computational tools for clustering analysis, motif analysis and epitope mapping will be integrated into the database.
